# From pineal photoreception to optogenetics: functional colour opponency based on a pineal bistable opsin

**DOI:** 10.1186/s40662-026-00485-1

**Published:** 2026-04-20

**Authors:** Seiji Wada, Emi Kawano-Yamashita, Mitsumasa Koyanagi, Akihisa Terakita

**Affiliations:** 1 Department of Biology, Graduate School of Science, Osaka Metropolitan University, Osaka, 558-8585 Japan; 2 Department of Chemistry, Biology, and Environmental Science, Faculty of Science, Nara Women’s University, Nara, 630-8506 Japan; 3 The OMU Advanced Research Institute for Natural Science and Technology, Osaka Metropolitan University, Osaka, 558-8585 Japan; 4 Present Address: Department of Chemistry and Biological Science, College of Science and Engineering, Aoyama Gakuin University, Sagamihara, 252-5258 Japan

**Keywords:** Opsin, Pineal organ, Colour opponency, Bistable nature, Optogenetics

## Abstract

Most animals sense light through opsins, which are photosensitive G protein–coupled receptors that utilize light information to regulate various physiological functions. In vertebrates, a representative example of such functions is vision, which is based on photoreception by visual opsins expressed in rod and cone photoreceptor cells of the retina. Accumulating photobiological evidence has gradually revealed the significance of the molecular properties of these opsins, as well as those of signal transduction–related molecules such as transducin, rhodopsin kinases, and arrestins, and their collective contribution to the functional characteristics of retinal photoreceptor cells. Furthermore, electrophysiological studies of animal photoreception have demonstrated that pineal-related organs in many vertebrates exhibit photosensitivity. Consequently, these organs are referred to as the “third eye,” in addition to the bilateral eyes. Light responses in pineal-related organs have been electrophysiologically described across various vertebrates, from lampreys to birds. In particular, species ranging from lampreys to lizards commonly exhibit two characteristic types of responses: “achromatic” and “chromatic.” Since the 1990s, genome projects have identified numerous opsin genes in diverse animal species and revealed their remarkable diversity. This has led to rapid advances in the study of “non-visual” opsins and their associated physiological functions. Along with these developments, the opsin repertoire in pineal-related organs has been shown to differ from that in the eyes. In this article, we review electrophysiological evidence of photosensitivity in pineal-related organs and describe the molecular basis underlying this sensitivity. Furthermore, we discuss the role of the pineal-specific opsin parapinopsin, which possesses molecular properties distinct from those of visual opsins, in photoreceptor cells, as well as examples of its application in optogenetics. Overall, this review highlights the molecular and physiological significance of pineal opsins and emphasises their emerging potential in optogenetic applications.

## Background

Most light-detection mechanisms in animals are mediated by opsins, which are typical G protein-coupled receptors (GPCRs) that activate G proteins in a light-dependent manner [1–4]. In general, this activation begins with the formation of the active state of the opsin through a conformational change triggered by the isomerization of the retinal chromophore from 11*-cis* to all*-trans* within the opsin upon light exposure. The activated G proteins subsequently drive a signal transduction cascade involving an effector enzyme, ultimately leading to cellular responses.

In rod and cone photoreceptor cells of the vertebrate retina—which underlie mesopic/scotopic and photopic vision, respectively—rhodopsin and cone opsins activate transducin (Gt). Activated Gt stimulates phosphodiesterase 6, which reduces the level of the second messenger cyclic guanosine monophosphate (cGMP). The resulting decrease in cGMP levels causes the closure of cyclic nucleotide-gated (CNG) channels, leading to hyperpolarization of photoreceptor cells. This hyperpolarization inhibits glutamate release from their synaptic terminals, modulating the transmission of light signals to retinal ganglion cells via bipolar cells [5]. Light-activated opsins are subsequently inactivated via phosphorylation by rhodopsin kinase and the binding of arrestin.

Previous evidence has established that rod and cone photoreceptor cells employ different types of signal transduction proteins. The molecular properties of opsins, together with those of the associated signal transduction molecules, partly determine the functional characteristics of photoreceptor cells. For example, rod cells exhibit higher sensitivity under mesopic and scotopic conditions, whereas cone cells display responses with higher temporal resolution under photopic conditions. However, emerging evidence indicates that light-response signals from rods and cones can interact at virtually every level of retinal signal processing, thereby making an important contribution to rod- and cone-mediated visual perception [6].

Although the mechanisms of phototransduction in rods and cones are well established, recent findings suggest that these mechanisms represent only one of several strategies in animals that have evolved for light detection. Beyond the retina, many vertebrates possess non-visual photoreceptive organs, such as pineal-related organs and deep brain regions, which employ distinct classes of opsins and signal transduction molecules. In particular, research on the pineal organ as a non-visual photoreceptive organ has a long history, and certain characteristic responses are shared among vertebrates. However, despite the identification of numerous pineal opsins, the molecular basis of pineal photoreception remains largely unresolved. Although neuronal light-responsive outputs in pineal-related organs were first reported more than 50 years ago, their physiological significance remains largely hypothetical. Herein, we review the repertoire and molecular characteristics of pineal opsins and their contributions to pineal photoresponses in vertebrate pineal-related organs. Furthermore, we discuss the optogenetic applications of a pineal bistable opsin, parapinopsin, in current research.

## Main text

### Diverse morphology and electrophysiological characteristics of vertebrate pineal-related organs

**Fig. 1:**
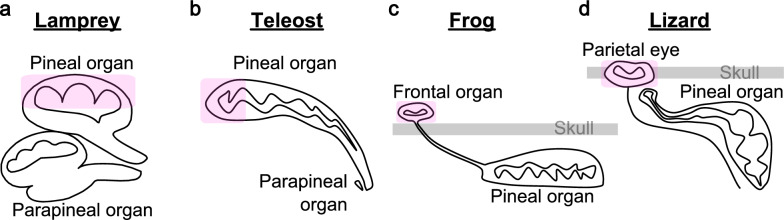
Schematic illustration of morphologically diversified pineal-related organs. Schematic representations of pineal-related organs in lampreys (**a**), teleosts (**b**), frogs (**c**), and lizards (**d**). Highlighted regions indicate areas that exhibit chromatic responses [11, 13–15, 20]

Vertebrate pineal-related organs exhibit considerable morphological diversity. Both lampreys and teleosts possess two pineal-related organs: the pineal and parapineal organs (Fig. 1a, b). In teleosts, the parapineal organ is considerably smaller than the pineal organ. By contrast, in lampreys, the pineal and parapineal organs are relatively large and comparable in size. Recently, the parapineal organ was reported to be incorporated into the left habenula of zebrafish during adulthood [7]. Among amphibians, frogs possess a frontal organ located on the skull alongside the pineal organ (Fig. 1c), and the nerve connecting the frontal organ to the brain passes through the skull. This frontal organ is believed to originate from a protrusion at the distal tip of the pineal organ [8, 9]. Among reptiles, lizards possess a parietal eye embedded in the skull—which is thought to arise from the development of the parapineal organ [8, 9]—in addition to the pineal organ (Fig. 1d). In avians, the pineal organ extends dorsally from the roof of the third ventricle and is located immediately beneath the skull [8]. In mammals, particularly in humans, the pineal organ is reduced to a small, cone-shaped structure situated near the center of the brain, between the two hemispheres and above the superior colliculus. The extraocular photosensitivity of pineal organs is thought to have been lost in mammals. This loss, together with the reduction in size, may reflect an evolutionary transition from direct photoreception to neuroendocrine regulation of circadian rhythms.

**Fig. 2:**
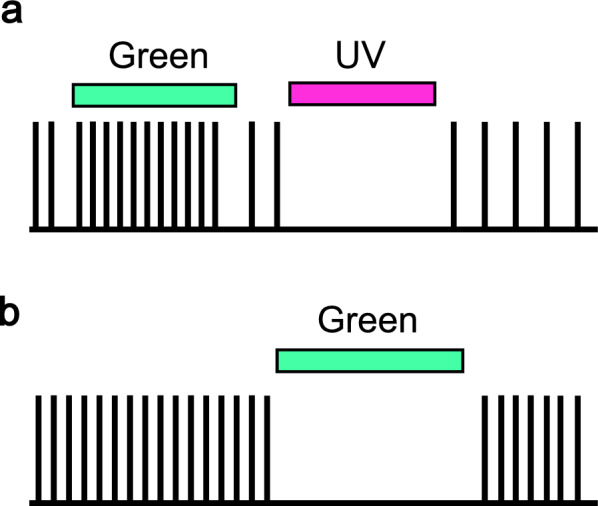
Schematic illustration of electrophysiological characteristics recorded in pineal-related organs. The chromatic response is characterised by the inhibition of neural firing in response to short-wavelength light and excitation in response to long-wavelength light (**a**). The achromatic response is defined as the inhibition of neural firing in response to visible light (**b**). UV, ultraviolet

Electrophysiological evidence that pineal-related organs are photoreceptive was first observed almost simultaneously in the parietal eye of lizards and frontal organs of frogs. Light-dependent neuronal activity has been recorded in the lizard parietal eye [10], and wavelength-dependent responses have been observed in the frontal organs of frogs [11]. The frontal organ in frogs exhibits two types of neural responses: chromatic and achromatic (Fig. 2a and b). The chromatic response is characterised by a decrease in neural firing in response to short-wavelength light and an increase in response to long-wavelength light. In frogs, the inhibitory component has a maximum sensitivity of approximately 355 nm, whereas the excitatory component peaks at approximately 515 nm. By contrast, the achromatic response involves the inhibition of neural activity in response to visible light, with maximum sensitivity between 560 and 580 nm [11]. Although the action spectra of these chromatic and achromatic responses vary across species, similar response patterns have been reported in the pineal organs of rainbow trout and northern pike [12, 13], the parietal eye of the lizard [14], and the pineal organ of the river lamprey [15]. This phenomenon, namely the light-dependent suppression of spontaneous firing in pineal ganglion cells, is thought to arise from the hyperpolarizing responses of pineal photoreceptor cells, which share similar signal transduction components with rods and cones [16, 17]. Similar to rods and cones, the principal pineal photoreceptors are believed to release glutamate in the dark and become hyperpolarized in response to light, thereby inhibiting glutamate release. Accordingly, pineal ganglion cells are presumed to express ionotropic glutamate receptors similar to those in retinal horizontal and some bipolar cells, and the suppression of neuronal firing by photoreceptor hyperpolarization is generally accepted as the primary mechanism. However, the molecular basis underlying neurotransmission between pineal photoreceptor and ganglion cells, including cell type-specific differences, remains largely unknown.

As our understanding of the neural characteristics of pineal-related organs has advanced, direct recordings of light responses from pineal photoreceptor cells have been performed. In the lamprey pineal organ, some photoreceptor cells exhibit visible light-induced hyperpolarization [18], whereas others respond to ultraviolet (UV) light [19]. These findings led to the hypothesis that two distinct types of photoreceptor cells generally underlie the pineal chromatic response. Interestingly, a landmark study demonstrated that in the lizard parietal eye, a single photoreceptor cell generates chromatic responses without involving other cells [20], highlighting an alternative mechanism of colour opponency in pineal-related organs.

### Molecular bases of photoreception in pineal-related organs

**Fig. 3:**
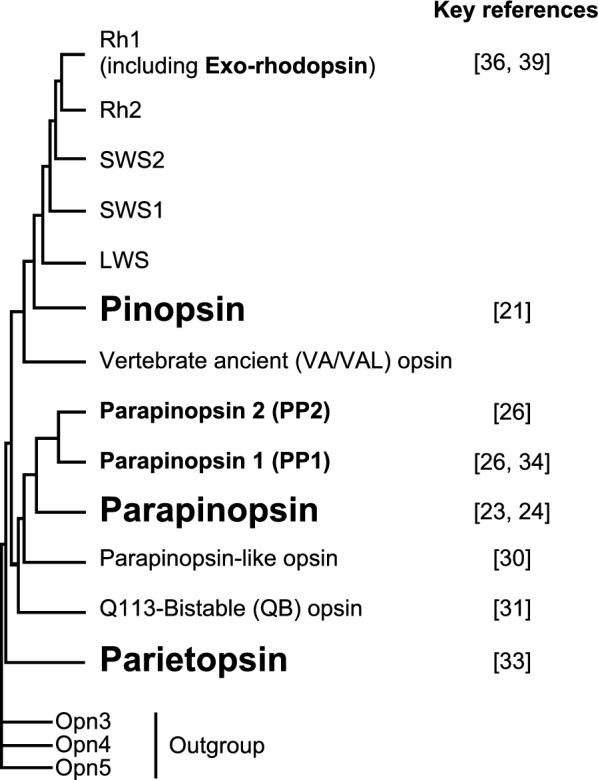
Phylogenetic relationships of vertebrate visual and non-visual opsins. Opsin groups highlighted in bold in the schematic molecular phylogenetic tree are generally specific to pineal-related organs, with key reference numbers indicated

Pinopsin, the first pineal-specific opsin, was identified in the pineal organs of chickens in 1994 (Fig. 3) [21]. Chicken pinopsin is a blue-sensitive opsin, and its absorption spectrum closely matches the action spectrum of the light-dependent acute inhibition of serotonin N-acetyltransferase, an enzyme responsible for regulating melatonin rhythms in chicken pineal organs [22]. Therefore, pinopsin-based photoreception may be involved in pineal endocrine function.

Parapinopsin was first identified in the pineal and parapineal organs of catfish in 1997 (Fig. 3) [23]. Subsequently, our group found parapinopsin and its expression in the pineal-related organs of river lamprey, rainbow trout, and iguana, all of which can discriminate between UV and visible light [24–26]. Our spectroscopic analyses further revealed that lamprey parapinopsin (LamPP) is UV-sensitive [24, 27]. These studies also demonstrated that parapinopsin has unique molecular properties distinct from those of visual opsins such as rhodopsin and cone opsins. Visual opsins form thermally unstable active states that rapidly decay and release retinal, whereas parapinopsins form thermally stable active states that are sensitive to visible light and do not release retinal. The photoproduct (active state), converted from the UV-sensitive dark (inactive) state, can revert to the original dark state. Because both states are stable, this property is referred to as “bistability”, which is similar to that observed in protostome (“invertebrate”) visual opsins, such as those of squid, spider, and *Drosophila* rhodopsins [1, 28], as well as coral opsins called cnidopsins [29]. Parapinopsin can be considered the first bistable opsin found in vertebrates. In lampreys and teleosts, parapinopsin couples with Gt [17]. Considering the UV-dependent hyperpolarization observed in pineal parapinopsin-expressing photoreceptor cells in lampreys [24], we can speculate that parapinopsin drives a Gt-mediated signal transduction cascade similar to that observed in rod and cone photoreceptor cells. We also reported that parapinopsin-related opsin (parapinopsin-like opsin), which is clearly distinguished from parapinopsin in the molecular phylogenetic tree, is blue-sensitive and expressed in the M5 nucleus of Schober in the deep brain of lampreys (Fig. 3) [30]. Q113-bistable (QB) opsin has been reported as an opsin present only in several lizards and tuatara [31, 32], exhibiting molecular properties similar to those of parapinopsin (Fig. 3) [31]. Furthermore, we have detected the QB opsin in iguanas (Accession No. AB626973, DDBJ database).

**Fig. 4:**
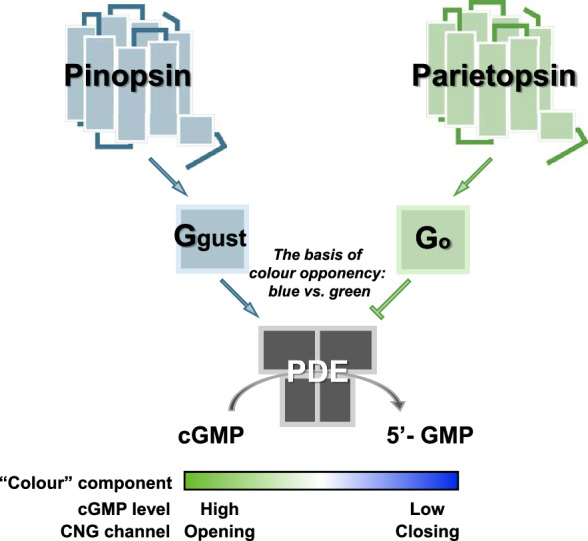
Mechanisms underlying the generation of colour opponency in the parietal eye of the side-blotched lizard. In side-blotched lizards, colour opponency is generated by antagonistic signal transduction involving two types of opsins: blue-sensitive pinopsin and green-sensitive parietopsin [33]. cGMP, cyclic guanosine monophosphate; CNG, cyclic nucleotide-gated; PDE, phosphodiesterase

Parietopsin was first identified as an opsin co-expressed with blue-sensitive pinopsin in the parietal eye of side-blotched lizards (Fig. 3) [33]. We subsequently identified parietopsin in the parietal eye of iguanas [25] and in the pineal organs of teleosts and lampreys [34, 35]. Parietopsin is green-sensitive, drives Go-mediated signal transduction, and induces depolarisation responses in a light-dependent manner [33, 35]. The co-expression of parietopsin and pinopsin in the side-blotched lizard contributes to the generation of colour opponency in single-photoreceptor cells of the parietal eye (Fig. 4). In iguanas, parietopsin is co-expressed with UV-sensitive parapinopsin [25]. Similar co-expression patterns have been observed in the pineal organs of zebrafish [34]. Furthermore, in lampreys, parietopsin and parapinopsin are expressed separately in both the pineal and parapineal organs [35], highlighting an intriguing aspect of the evolutionary process underlying the co-expression and repertoire of non-visual opsins. In addition, we observed a significantly higher number of parietopsin-expressing cells in the parapineal organ than in the pineal organ, suggesting potential functional diversification between the two organs in lampreys.

In addition to the UV-sensitive parapinopsin (parapinopsin 1, PP1), teleosts possess blue-sensitive parapinopsin 2 (PP2), which originated from teleost-specific whole-genome duplication (Fig. 3) [26]. We found that spectral tuning between PP1 and PP2 is achieved by substituting amino acid residues in helix II; however, the spectral tuning mechanism of teleost parapinopsins differs from that of vertebrate SWS1 opsins and fruit fly UV-sensitive visual opsins. Our group previously found that PP2 is expressed in pineal photoreceptor cells containing serotonin, a precursor of melatonin. Recent reports indicate that although pinopsin genes are present in Atlantic and Indo-Pacific tarpon within the Elopiformes of the Elopomorpha, virtually all other teleosts have lost pinopsin [31, 36, 37]. Therefore, teleost-specific PP2 may play a similar role in the regulation of melatonin synthesis and secretion. PP2 is expressed in pineal photoreceptor cells in a pattern that is mutually exclusive with PP1. Collectively, the spectral difference between UV-sensitive PP1 and blue-sensitive PP2, resulting from amino acid substitutions, may have facilitated neofunctionalization [26].

Visual pigments such as rhodopsin and long-wavelength-sensitive (LWS) cone opsin are also expressed in pineal-related organs. In the pineal organs of lampreys, rhodopsin and LWS opsin are localized on the ventral side, while LWS opsin is additionally expressed on the caudal side of the pineal stalk [38]. In teleosts, zebrafish express LWS opsin in the pineal organ, where it is co-expressed with PP2 [26]. Exo-rhodopsin was first identified in the pineal organs of zebrafish (Fig. 3) [39]. During the evolution of the rhodopsin gene, a retro-duplication resulted in the generation of an intron-less “new” rhodopsin, while the original intron-containing gene was retained. Teleosts utilize this new rhodopsin for scotopic vision in their eyes, whereas the “old” rhodopsin (i.e., exo-rhodopsin) is used for pineal photoreception [36].

### Colour opponency generated within single-photoreceptor cells in pineal-related organs

Our previous studies have demonstrated that, in lampreys, pineal colour opponency between UV and visible light is generated with two types of pineal photoreceptor cells, UV-sensitive parapinopsin-expressing cells and green-sensitive parietopsin-expressing cells [35]. This mechanism is similar to the cellular basis of vertebrate colour vision, which relies on the use of multiple types of photoreceptor cells. However, colour opponency can be achieved within a single-photoreceptor cell in the parietal eye of the side-blotched lizard [20]. Specifically, the co-expression of blue-sensitive pinopsin and green-sensitive parietopsin activates gustducin and Go, respectively, in a light-dependent manner. These distinct G protein pathways antagonistically regulate the opening and closing of CNG channels through blue light–induced decreases and green light–induced increases in cGMP levels (Fig. 4) [33]. Unlike the lamprey pineal system, the conventional view that colour opponency requires multiple types of photosensitive cells, such as cone photoreceptors in vertebrate colour vision, does not apply to the parietal eye system. Furthermore, we discovered an even simpler system for generating colour opponency in the zebrafish pineal organs that does not require multiple types of opsins [34].

**Fig. 5:**
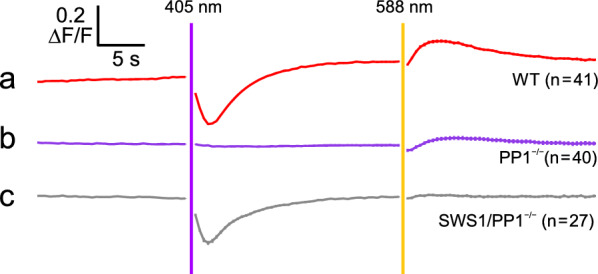
Parapinopsin 1 (PP1) alone generates chromatic calcium responses. Changes in calcium levels upon 405- and 588-nm light stimulation in PP1-expressing cells of wild-type (WT) (**a**, red; n = 41), PP1-deficient (PP1^−/−^) fish (**b**, purple; n = 40), and SWS1 opsin–expressing/PP1-deficient (SWS1/PP1^−/−^) fish (**c**, grey; n = 27). Error bars represent the standard error of the mean. Light intensities for the 405- and 588-nm stimuli were approximately 3.2 × 10^14^ and 5.4 × 10^17^ photons/cm^2^/s, respectively. Both stimuli were applied for approximately 450 ms. ∆F/F values were normalised to the average of 10 data points collected prior to the initial light stimulation. Adapted from reference [28] and licenced under the Creative Commons Attribution 4.0 (CC BY 4.0) licence

To investigate the functional significance of the bistable nature of parapinopsin, we used two-photon imaging to analyse calcium responses in PP1-expressing photoreceptor cells of *Tg(pp1:GCaMP6s)* zebrafish, in which the fluorescent calcium indicator GCaMP is expressed specifically in PP1-expressing cells [34]. In the pineal organ of zebrafish, PP1 is co-expressed with green-sensitive parietopsin. We found that photoreceptor cells expressing both PP1 and parietopsin exhibit chromatic calcium responses, characterised by a decrease in intracellular calcium levels in response to UV light and an increase in response to visible light (Fig. 5a). These observations suggest that colour opponency is mediated by PP1 (rather than pinopsin), together with parietopsin, through a mechanism similar to that observed in the parietal eye of the side-blotched lizard. To elucidate the mechanisms underlying these calcium responses, we examined knockout zebrafish lines and found that PP1-deficient mutant fish do not respond to either UV or visible light, indicating that PP1 alone is necessary and sufficient to generate chromatic calcium responses (Fig. 5b).

**Fig. 6:**
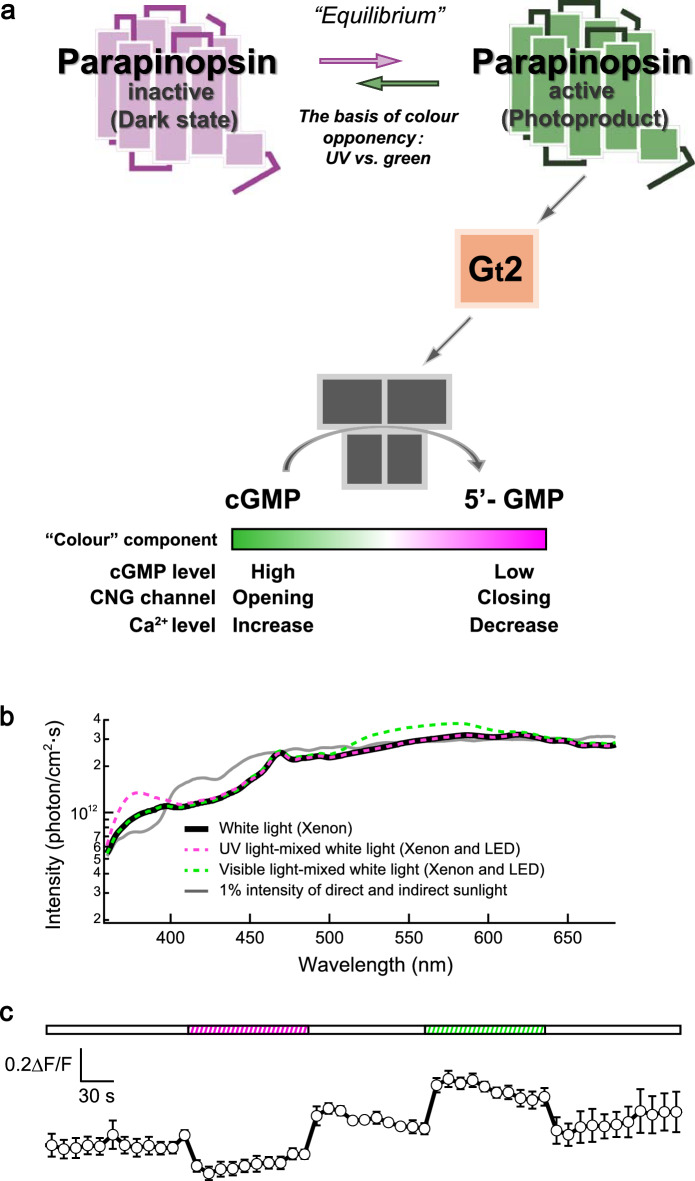
Colour response based on the mechanism generating a photoequilibrium between the two states of parapinopsin 1 (PP1) under physiological light conditions.** a** Schematic diagram illustrating the mechanism of colour opponency based on photoreception by PP1 alone. PP1 forms an equilibrium between a UV-sensitive inactive state and a visible light-sensitive active state, and shifts in this equilibrium generate color opponency. **b** Spectra of the xenon white light (solid black trace), ultraviolet (UV) light-emitting diode (LED) light–mixed white light (broken magenta trace), and visible LED light–mixed white light (broken green trace) are compared with those of 1% intensity of direct sunlight (solid grey trace), measured at 2:30 p.m. on April 16, 2018, in Osaka, Japan. **c** Changes in calcium levels in PP1-expressing cells following prolonged exposure to white light, with or without UV or visible LED light. The durations of exposure to UV and green LED light–mixed white lights were approximately 100 s. The light intensities of UV and LED lights used for the exposures were approximately 1.3 × 10^13^ and 7.6 × 10^13^ photons/cm^2^/s, respectively. White light intensity was approximately 8.0 × 10^14^ photons/cm^2^/s (360–780 nm). ΔF/F values were normalised to the average of 10 data points between UV and visible light stimuli. Error bars represent the standard error of the mean (n = 5). Notably, the relaxation of responses to UV and green LED light was not observed owing to ‘time-lapse measurement’ with low temporal resolution. Adapted from reference [28] and licenced under the Creative Commons Attribution 4.0 (CC BY 4.0) licence. cGMP, cyclic guanosine monophosphate; CNG, cyclic nucleotide-gated

The presence of “background” light is the key underlying the mechanism by which PP1 induces chromatic calcium responses in single cells. During two-photon time-lapse imaging, the 930-nm two-photon laser, which excites GCaMP and is equivalent to blue light (approximately 465 nm) in the one-photon context, illuminates PP1 as background light. This generates an equilibrium-like state between the dark (UV-sensitive) and active (visible light-sensitive) states of PP1 (Fig. 6a). The coexistence of sufficient amounts of these two states enables chromatic calcium responses by altering the extent of Gt2 activation in accordance with shifts in the equilibrium between them. Specifically, calcium levels decrease in response to UV light stimulation and increase in response to visible light stimulation, mediated through the respective increases and decreases in the active-state ratio at equilibrium. To confirm that the chromatic calcium responses are based on the bistable property of PP1, we generated a “bistable-to-monostable opsin” zebrafish mutant (SWS1/PP1^−/−^), in which the UV-sensitive and Gt-coupled visual opsin SWS1—which is not bistable—is expressed in place of PP1 in the photoreceptor cells that normally express PP1. We subsequently observed a clear decrease in calcium levels in response to UV light but no increase in response to visible light (Fig. 5c). This finding supports the notion that chromatic responses are based on the bistable nature of PP1. Notably, although the dark state of SWS1 is continuously bleached by background light owing to its photobleaching properties, the clear response to UV stimulation is likely attributable to the pineal organ possessing an 11-*cis*-retinal supply mechanism similar to that of the retina.

“Opponent” responses based on bistability have been observed in the photoreceptor cells of white-eyed *Drosophila* [40]. However, these responses have only been observed under specific experimental conditions. Therefore, whether the phenomena observed under a two-photon laser background also occur in natural settings remains unclear. To explore this possibility, we examined whether wavelength-dependent photoequilibria could be formed using the purified PP1 protein. We found that different ratios of the two PP1 states are formed under different light environments with varying spectral distributions of light. As expected, the ratio of the PP1 dark state to its active state differs between sunny areas (enriched in long-wavelength light) and shady areas (enriched in short-wavelength light) in the late afternoon [34]. Accordingly, we performed “physiological” two-photon imaging using xenon white light, which has a spectral distribution approximately 1% of the intensity of direct sunlight (Fig. 6b). The results showed that colour-dependent responses are elicited by slight differences in the spectral components of light in PP1-expressing cells (Fig. 6c). These results suggest that PP1-based colour opponency operates under natural light conditions [34].

To establish stable photoequilibria between the dark inactive state and active photoproduct, the inactivation of active photoproduct by regulatory molecules, such as kinases and arrestins, should be considered. This is because a sufficient amount of the PP1 active photoproduct that is not inactivated must be maintained to permit visible light-induced increases in calcium levels. To investigate the inactivation mechanism of PP1 active photoproduct, we focused on its regulatory components. We have previously identified GRK7a as the major GPCR kinase in PP1-expressing cells under weak light stimulation [41]. Additionally, we detected two types of arrestins, Arr3a and Sagb, in PP1 cells. Interestingly, Arr3a plays a major role in terminating the PP1-mediated response under weak light conditions, whereas Sagb becomes the dominant arrestin under strong light conditions. PP1-mediated responses to strong light are terminated more slowly by Sagb [42]. This light intensity-dependent switching of arrestins is thought to be crucial for maintaining sufficient levels of the “alive” active state of PP1 under strong light conditions, where photoequilibria are formed. Furthermore, this arrestin switching suggests that a single PP1 cell can switch between luminance detection and colour detection modes depending on the light intensity. Notably, we found that, in the lamprey pineal organ—where parapinopsin is distributed to photoreceptor cells exclusively from parietopsin-expressing cells—parapinopsin employs a single type of arrestin: β-arrestin [16].

The colour-detection mechanism we identified in a single PP1-expressing cell, which relies on a single opsin, raises an intriguing question regarding the functional properties, including advantages and limitations, of this single-cell, single-opsin system when compared with the multichromatic visual system of the eye, which depends on multiple opsins and cell types. For instance, in trichromatic vision, colour perception is represented in a three-dimensional space defined by luminance (R + G), red–green opponency (R–G), and blue–yellow opponency ((R + G)–B), where R, G, and B correspond predominantly to signals derived from long-, medium-, and short-wavelength-sensitive cones, respectively [43]. By contrast, the colour information output of PP1 is determined unidimensionally because it originates from a single cell expressing a single opsin. Consequently, the conditions in which both UV and visible light intensities are low, as well as those in which both are high, may yield similar output levels. This suggests that colour detection by PP1-expressing cells alone may not encode luminance information. Nevertheless, the expression of Arr3a and Sagb, two types of arrestins that function at different light intensities in PP1-expressing cells, may enable switching between distinct operational modes depending on light intensity: detecting brightness under dim light through UV sensitivity and detecting colour under brighter conditions [42]. Despite the limited detectable range of light intensity, such a mechanism may allow a single cell to dynamically alternate between luminance and chromatic detection modes.

Taken together, these characteristics suggest that the dynamic range of light and colour detection in PP1-expressing cells is narrower than that of the retina. In addition, while the human retinal colour vision represents brightness and chromatic information along separate dimensions, the single-cell photoreceptive system of PP1-expressing cells encodes both types of information along a single dimension. Therefore, although this mechanism does not require a sophisticated neural network and may be advantageous from a biological cost perspective, it has a limited dynamic range that likely imposes a trade-off. Moreover, within the intensity range that permits colour detection, cone photoreceptors exhibit rapid signal termination, whereas PP1-expressing cells respond more slowly. The temporal resolution of colour detection in PP1-expressing cells may be considerably lower, potentially reflecting an adaptation to slow, sustained light responses rather than rapid visual processing.

The functional significance of neuronal light information output from pineal photoreceptor cells, including PP1-expressing cells, remains unclear. A key consideration for future studies is that the colour information output based on the molecular properties of PP1 requires a certain level of background illumination and is expected to have a lower temporal resolution than that of retinal cones. In this context, pineal cells, particularly PP1-expressing cells, may respond to gradual light changes during the day, such as those caused by moving clouds, thereby contributing to behavioural outputs. Alternatively, if the pineal organ possesses any spatial resolving ability, it may be able to extract directional information from skylight patterns. Further investigation is required to assess these possibilities.

### Development of parapinopsin as a tool for reversible optical control of GPCR signalling

Microbial rhodopsins have been widely adopted as optogenetic tools, and significant progress has been made in their optimization. However, despite the development of OptoXRs [44], there remains a strong demand for novel animal opsin-based tools capable of optogenetically controlling intracellular signalling via GPCRs. Our group was among the first to explore the potential of several animal opsins as optogenetic tools [45–47].

We have previously demonstrated that intracellular cyclic AMP levels in HEK293 cells expressing LamPP can be regulated in a wavelength-dependent manner [17]. In these cells, LamPP drives the Gi/o signalling cascade in response to UV light. Specifically, a flash of UV light induces a sustained decrease in cyclic AMP levels, whereas a subsequent flash of visible light—which restores LamPP to its original dark state—leads to the recovery of cyclic AMP levels. These findings indicate that LamPP may serve as a useful tool for the light-dependent regulation of intracellular signal transduction. LamPP (referred to as UVLamP) has also been reported to selectively couple to Gi/o proteins and exhibit robust, reversible signalling in GIRK currents, as measured using whole-cell patch-clamp techniques [48]. In recent years, LamPP has emerged as a promising optogenetic tool for precise and reversible manipulation of GPCR signalling cascades in vivo.

LamPP has been applied across diverse systems to achieve reversible and wavelength-dependent control of biological processes such as neuronal activity. When introduced into hypothalamic neurones and retinal ON-bipolar cells, LamPP (referred to as Lamplight) enables bidirectional optical modulation of neuronal activity and visual responses through alternating short- and long-wavelength illumination. In a degenerative retinal model, LamPP successfully restores light sensitivity, suggesting its potential therapeutic utility in both central and sensory neural circuits [49]. Another research group has used LamPP (referred to as PPO) in mammalian neurones to develop an optogenetic strategy for rapid and reversible presynaptic inhibition [50]. They developed a method to activate LamPP using blue light (approximately 470 nm), a wavelength commonly used in optogenetic experiments. In their system, LamPP is activated by blue light (470 nm), and deactivation is achieved using amber light (approximately 590 nm), enabling the suppression of glutamate, gamma-aminobutyric acid, and dopamine release from synaptic terminals. Notably, they demonstrated time-locked modulation of reward-related behaviour in vivo, highlighting the potential of LamPP as a tool for behavioural neuroscience [50]. Together with our group, Hagio et al. demonstrated that LamPP is effective in neurones and non-neuronal tissues, such as cardiomyocytes in zebrafish. They showed that LamPP enables the reversible suppression of cardiac contractions and motor outputs, thereby expanding its application to organ-level physiological control with high optical precision [51].

These robust wavelength controls and Gi/o-specific signal transduction properties of LamPP represent clear advantages for its use as an optogenetic tool, although challenges remain regarding the activation of other G proteins and the retinal supply system. The robustness of wavelength-dependent control achieved with LamPP is attributable to the separation of more than 100 nm between the absorption maxima of its dark- and light-activated states. From this perspective, we discuss the optogenetic potential of other bistable animal opsins.

Mosquito Opn3, whose molecular properties we first characterized [52], has emerged as a widely used optogenetic tool for GPCR manipulation, comparable to parapinopsin [53, 54], although the molecular properties of mammalian Opn3 still remain largely unknown. Mosquito Opn3 can utilize *13-cis* retinal, which is produced through thermal isomerization from all*-trans* retinal, as its chromophore, thereby providing high versatility across cell types in terms of retinal supply. This represents an advantage not shared by LamPP. In *Caenorhabditis elegans*, the optogenetic potential of Opn3 under all*-trans* retinal conditions is more than one thousand times greater than that of bovine rhodopsin [55]. Because the spectral shift between the dark and photoproduct states of Opn3 is relatively small, wavelength-dependent control based on its bistability is not expected. However, chimeric Opn3, constructed by replacing its intracellular loop with that of an adrenergic receptor and capable of activating Gs or Gq, holds considerable promise as an optogenetic actuator. By contrast, we generated a Gs-coupled LamPP variant by replacing its intracellular loop; however, a Gq-coupled version has not yet been reported.

Members of the Opn5m group, which includes mammalian Opn5, exhibit spectral characteristics of their dark- and light-activated states similar to those of LamPP [56–58], and their potential utility has been discussed in several optogenetic studies [59, 60]. To the best of our knowledge, unlike LamPP, no reports have demonstrated such pronounced wavelength-dependent ON/OFF control of cellular activities using Opn5m in vivo, although such control has been demonstrated in vitro. However, the fact that Opn5m, but not LamPP, activates Gq [48, 60] may be advantageous for expanding the potential applications of Opn5m. Notably, because Opn5m shows high selectivity for G_14_ and can activate Gi, Gq, G_11_, and G_15_ [56–58, 61], careful consideration of the G protein repertoire in the target cell type is required. In addition, the newly identified bistable opsin QB opsin exhibits an absorption spectrum similar to that of LamPP [31], suggesting its potential applicability for optogenetic applications.

**Fig. 7:**
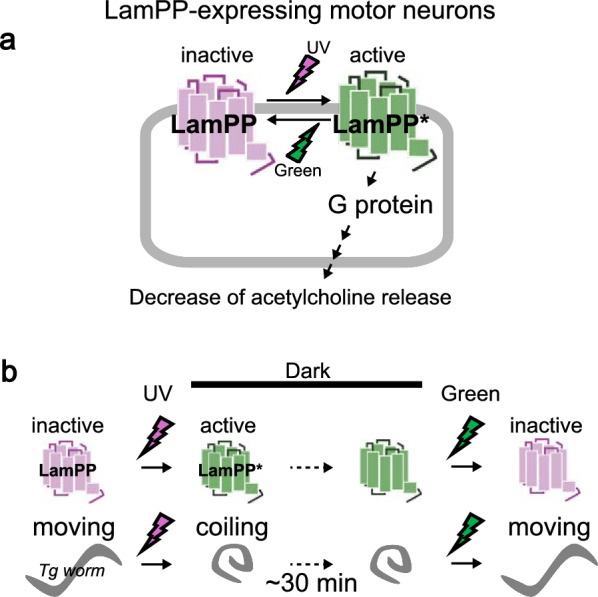
Drastic and sustained responses evoked by lamprey parapinopsin (LamPP) in *Caenorhabditis elegans.* **a** Schematic representation of LamPP-expressing motor neurones in transgenic worms. Ultraviolet (UV) stimulation decreases acetylcholine release in neurones, whereas green light—which returns LamPP from active states to its original inactive states—restores acetylcholine release. **b** Behavioural responses to UV and green light in transgenic worms

Recently, our group applied LamPP to *Caenorhabditis elegans* and demonstrated colour-controlled modulation of worm behaviour via Gi/o signalling in vivo using UV and green light [55]. Specifically, we generated transgenic worms expressing LamPP in cholinergic motor neurones under the *unc-17* promoter (Fig. 7a). These transgenic worms cease movement and exhibit coiling behaviour in response to a 5-s UV flash. Interestingly, the coiling persists for 30 min, and movement is restored upon subsequent green light illumination (Fig. 7b). This remarkably prolonged response to light is attributed to the exceptional stability of the active state of LamPP, making it ideal for applications in which continuous light exposure is undesirable. We have confirmed the reliability of LamPP as a behavioural manipulation tool across species [51, 55]. Collectively, these studies highlight the development of LamPP from a photobiochemical curiosity to a versatile and robust optogenetic tool. With its combination of wavelength-dependent reversibility based on bistability and high biological compatibility, LamPP-based optogenetics emerge as a promising core technology for future research into neural circuitry, organ-level physiology, and GPCR-targeted therapeutic strategies.

## Conclusions

The discovery of diverse opsins and the characterisation of their molecular properties have revealed physiologically intriguing phenomena associated with the functional characteristics of the pineal organ. By contrast, apart from light-regulated endocrine functions such as melatonin secretion, no direct evidence is available regarding how pineal-related organs utilise the light information they receive. This remains a major unresolved question in the field of photobiology. Notably, the bistability-based, wavelength-dependent reversibility of parapinopsin, which is strongly linked to significant physiological relevance, has recently begun to be applied in optogenetics. This development represents a new direction in GPCR-based optogenetics and holds considerable potential as a foundation for therapeutic strategies targeting GPCRs. This review summarizes recent advances and highlights the emerging physiological and optogenetic significance of pineal-related opsins.

## Data Availability

Not applicable.

## Abbreviations

GPCRs: G protein-coupled receptors
Gt: Transducin
cGMP: Cyclic guanosine monophosphate
CNG channel: Cyclic nucleotide-gated channel
PP1: Parapinopsin 1
PP2: Parapinopsin 2
LWS opsin: Long-wavelength-sensitive cone opsin
LamPP: Lamprey parapinopsin
QB opsin: Q113-bistable opsin
